# Outcomes in Brugada Syndrome Patients With Implantable Cardioverter-Defibrillators: Insights From the SGLT2 Registry

**DOI:** 10.3389/fphys.2020.00204

**Published:** 2020-03-10

**Authors:** Sharen Lee, Ka Hou Christien Li, Jiandong Zhou, Keith Sai Kit Leung, Rachel Wing Chuen Lai, Guoliang Li, Tong Liu, Konstantinos P. Letsas, Ngai Shing Mok, Qingpeng Zhang, Gary Tse

**Affiliations:** ^1^Laboratory of Cardiovascular Physiology, Li Ka Shing Institute of Health Sciences, Hong Kong, China; ^2^Faculty of Medical Sciences, Newcastle University, Newcastle upon Tyne, United Kingdom; ^3^School of Data Science, City University of Hong Kong, Hong Kong, China; ^4^Department of Cardiovascular Medicine, The First Affiliated Hospital of Xi’an Jiaotong University, Xi’an, China; ^5^Tianjin Key Laboratory of Ionic-Molecular Function of Cardiovascular Disease, Department of Cardiology, Tianjin Institute of Cardiology, Second Hospital of Tianjin Medical University, Tianjin, China; ^6^Laboratory of Cardiac Electrophysiology, Second Department of Cardiology, Evangelismos General Hospital of Athens, Athens, Greece; ^7^Department of Medicine and Geriatrics, Princess Margaret Hospital, Hong Kong, China; ^8^Department of Cardiology, The First Affiliated Hospital of Dalian Medical University, Dalian, China; ^9^Xiamen Cardiovascular Hospital, Xiamen University, Xiamen, China

**Keywords:** Brugada syndrome, ICD (implantable cardioverter-defibrillator), sudden cardiac death, ventricular tachiarrhythmias, risk stratificacion

## Abstract

**Background and Objectives:**

Brugada syndrome (BrS) is a cardiac ion channelopathy with characteristic electrocardiographic patterns, predisposing affected individuals to sudden cardiac death (SCD). Implantable cardioverter-defibrillator (ICD) is used for primary or secondary prevention in BrS, but its use remains controversial amongst low-risk asymptomatic patients. The present study aims to examine indicators for ICD implantation amongst BrS patients with different disease manifestations.

**Methods:**

This study included BrS patients who received ICDs between 1997 and 2018. The cohort was divided into three categories based on presentations before ICD implantation: asymptomatic, syncope, ventricular tachycardia/ventricular fibrillation (VT/VF). Univariate and multivariate Cox-regression analysis were performed to identify independent predictors of appropriate and inappropriate shock delivery.

**Results:**

A total of 136 consecutive patients were included with a median follow-up of 95 (IQR: 80) months. Appropriate shocks were delivered in 34 patients (25.0%) whereas inappropriate shocks were delivered in 24 patients (17.6%). Complications occurred in 30 patients (22.1%). Type 1 Brugada pattern were found to be an independent predictor of appropriate shock delivery, whilst the presence of other arrhythmia was predictive for both appropriate and inappropriate ICD shock delivery under multivariate Cox regression analysis.

**Conclusion:**

ICD therapy is effective for primary and secondary prevention of SCD in BrS. Whilst appropriate shocks occur more frequently in BrS patients presenting with VT/VF, they also occur in asymptomatic patients. Further research in risk stratification can improve patient prognosis while avoid unnecessary ICD implantation.

## Introduction

Brugada syndrome (BrS) is a cardiac ion channelopathy with characteristic electrocardiographic Brugada patterns (BrP) in the right precordial leads (Antzelevitch et al., 2005). Patients suffering from BrS are at an elevated risk of developing syncope, ventricular tachy-arrhythmias and sudden cardiac death (SCD), often in an absence of overt structural abnormalities, although fibrosis in the right ventricle and the right ventricular outflow tract (RVOT) has been reported. Patients with BrPs are diagnosed with BrS based on criteria including symptoms, inducible ventricular tachy-arrhythmias during electrophysiological studies (EPS), or with a family history of SCD or positive genetic findings (Antzelevitch et al., 2005).

Currently, implantable cardioverter-defibrillators (ICDs) is the only definitive treatment with well-demonstrated efficacy in the management of SCD amongst BrS patients (Brugada et al., 2000). Whilst those who are symptomatic or manifest with spontaneous type 1 patterns are recognized to be at higher risks and should receive ICDs, whether asymptomatic patients should have device therapy remains controversial. Reliable predictors for SCD risk stratification of BrS patients are yet to be available. For example, the predictive value of ventricular tachy-arrhythmia inducibility in EPS remains debated when results were examined in large registries, with significantly greater event rates for inducible arrhythmias when compared to spontaneously occurring VT/VF or SCD (Brugada et al., 2002; Priori et al., 2012). Electrocardiographic indices may be of predictive value but are limited by the dynamic nature of conduction or repolarization abnormalities (Tse et al., 2017; Deliniere et al., 2019). Although BrS is found to be associated with genetic mutations, notably in the SCN5A gene, the low prevalence of genetic testing (∼25%) and heterogeneity in patients’ genetic profile restricts its application in risk stratification (Milman et al., 2018; Chen C. et al., 2019).

The decision on ICD implantation is further complicated by the comparable rates of appropriate shock delivery and complication, including inappropriate shock delivery, device or lead malfunction and infection (Sacher et al., 2013; Conte et al., 2015). Whilst episodes of appropriate shock can serve as surrogates for SCD, which demonstrates the therapeutic value of ICD, the potentially significant complication rate should also be noted. Therefore, it is important to gain insights on the outcome and risk-factors of both appropriate shocks and complications amongst different patient subgroups to improve patient prognosis, whilst avoid unnecessary ICD implantation. The present study investigated the outcomes of BrS patients receiving ICDs for primary or secondary prevention of SCD to examine indicators for ICD implantation amongst BrS patients presented with ventricular tachy-arrhythmia, syncope or asymptomatic.

## Methods

### Study Population

This retrospective study received Ethics approval from The Joint Chinese University of Hong Kong – New Territories East Cluster Clinical Research Ethics Committee and is based on datasets that have already been made available in an online repository (Tse et al., 2019a, b, c). The study inclusion criteria include: (1) spontaneous, fever or drug-induced presentation of type 1 or 2 BrS; (2) undergone ICD implantation between 1997 and 2018. Type 1 BrP is defined as a coved-shape ST segment with elevation of >2 mm followed by a negative T-wave, and type 2 pattern is defined as convex ST segment with >0.5 mm elevation followed by variable T-wave, resulting in a saddleback-shaped morphology (Antzelevitch et al., 2005). The diagnosis of BrS is made based on the 2017 ACC/AHA/HRS Guideline (Shen et al., 2017), after reviewing documented patient history, and confirmed by analysis of all documented ECG by S.L. and G.T.

The cohort was divided into three groups based on patient history prior to ICD implantation: (1) asymptomatic; (2) syncope; (3) ventricular tachycardia/ventricular fibrillation (VT/VF). Asymptomatic was defined as the absence of syncope and VT/VF manifestation, whilst the syncope and VT/VF group was categorized based on the patient presentation of the respective symptoms. Patients presented with both syncope and VT/VF were included in both groups.

### Patient Data

Clinical data was extracted from manual review of case records. The following baseline clinical data was collected: (1) sex; (2) age of initial BrP presentation; (3) family history of BrS and SCD/VF; (4) type of BrP presented, presence of evolution and fever; (4) presence of other arrhythmias [supraventricular tachycardia (SVT) and ectopic beats (SVE), atrial fibrillation (AF), and tachycardia (AT), atrial flutter]; (5) inducibility in electrophysiological study (EPS); (6) abnormalities in 24-h Holter; (7) presentation of syncope and VT/VF; (8) follow-up period; (9) quinidine use (dosage and duration); (10) baseline average ECG parameters recorded on the first available ECG. Follow-up period is defined as the period from initial presentation of BrP to patient’s death, time of contact loss, or until 22th April 2019. Patients are considered to present type 1 BrP if type 1 pattern is presented initially, or at any point in follow-up. Evolution of ECG pattern is defined by the change of BrP type, or resolution of pattern, during continuous follow-up. The baseline average ECG parameters include: (1) ventricular rate; (2) P wave duration; (3) PR interval; (4) QRS interval; (5) QT interval; (6) QTc interval; (7) P wave axis; (8) QRS axis; (9) T wave axis; (10) lead V1 S wave amplitude; (11) lead V5 R wave amplitude.

Furthermore, the following details on ICD implantation was collected: (1) date and type of initial and subsequent ICD implantation; (2) number of appropriate and inappropriate shocks; (3) reason of inappropriate shocks; (4) experience of lead malfunction, dislocation, and infection; (5) number of VT/VF episodes in total, prior to and after ICD implantation. Appropriate shocks are defined as shocks delivered to correct sustained VT/VF, whilst inappropriate shocks are shocks delivered for other reasons. Device setting to determine sustained VT/VF was based on guidelines at the time of device implantation, and subject to further adjustment given the patient’s condition. Episodes of VT/VF include both symptomatic and asymptomatic events detected by the ICD. Lead malfunction includes lead failure and fracture that required surgical extraction or replacement.

### Statistical Analysis

All statistical analysis was performed using Stata MP13. For clinical characteristics, categorical variables were compared between the three groups using Fisher’s exact test, whilst continuous variables were compared by Kruskal–Wallis one-way ANOVA. For baseline characteristics and ICD outcomes with significant inter-group differences, the continuous variables were compared using Dunn’s pairwise comparison test with Sidak correction, and the categorical variables undergone pairwise comparison by Fisher’s exact test again for further examination. Appropriate shock-free survival was estimated by the Kaplan–Meier survival curve and compared through the log-rank test. To examine the individual contribution of clinical characteristics to the execution of appropriate shocks during follow-up, univariate Cox proportional hazard ratio regression with Efron’s method for ties was performed on the entire cohort, and the three subgroups.

Variables with *P*-value < 0.05 in univariate analysis were then selected into the multivariate Cox regression model for the prediction of appropriate shock. The analyzed clinical features include: (1) sex; (2) age of initial BrP presentation; (3) presence of pattern evolution; (4) presentation of type 1 BrP; (5) fever-induced pattern; (6) presentation of syncope; (7) presence of other arrhythmia; (8) inducible EPS. Similarly, the Cox regression was used to examine the effect of the following clinical features on the occurrence of inappropriate shock: (1) age at which BrP ECG manifested; (2) evolving ECG pattern; (3) syncope; (4) other cardiac arrhythmias. Statistical significance is defined as *P*-value < 0.05.

## Results

### Baseline Characteristics

The present cohort included 136 Han Chinese BrS patients receiving ICDs (mean age of initial BrP presentation: 50.0 ± 15.4 years, male: 96.3%, spontaneous type 1 pattern: 64.7%). Their baseline characteristics of the cohort categorized into asymptomatic (*n* = 26, 19.1%), syncope (*n* = 99, 72.8%), VT/VF (*n* = 36, 26.5%) groups are summarized in Table 1A. Of the 136 patients, 25 patients suffered from both syncope and VT/VF. ICD implantation took place for both primary prevention amongst patients who were asymptomatic or only presented with syncope (*n* = 100, 73.5%) or secondary SCD prevention in patients presented with VT/VF (*n* = 36, 19.1%). ICD implantation in asymptomatic patients were performed based on VT/VF inducibility in EPS (*n* = 16), and a combination of positive drug challenge test and family history of SCD (*n* = 1) (Priori et al., 2013). The remaining devices were implanted at the patient’s request after discussion with the attending physician. Amongst patients with type 1 BrP, 78 patients (88.6%) presented with type 1 pattern initially, and for 10 patients (11.4%) the pattern resolved or evolved into type 2 during follow-up. Other types of arrhythmia were presented in 35 patients (25.7%), notably SVT (*n* = 10), SVE (*n* = 3), AF (*n* = 10), and AT (*n* = 4) and atrial flutter (*n* = 3). 4 (2.94%) and 14 (10.3%) patients have a family history of BrS and SCD/VF, respectively.

**TABLE 1A:** Baseline characteristics.

Feature	Overall (*n* = 136)	Asymptomatic (*n* = 26)	Syncope (*n* = 99)	VT/VF (*n* = 36)	*P*-value
Male	131 (96.3)	26 (100)	107 (96.0)	35 (97.2)	0.827
Age at Initial BrP presentation	50.0 ± 15.4	49.0 ± 13.8	50.8 ± 14.6	47.6 ± 19.0	0.606
Type 1 BrP	88 (64.7)	15 (57.7)	65 (65.7)	26 (72.2)	0.496
BrP evolution	43 (31.6)	8 (30.8)	32 (32.3)	11 (30.6)	1.00
Fever-induced type 1 BrP	7 (5.15)	0 (0)	5 (5.05)	3 (8.33)	0.385
Family history of BrS	4 (2.94)	2 (7.69)	2 (2.02)	0 (0)	0.158
Family history SCD	14 (10.3)	5 (19.2)	9 (9.09)	0 (0)	**0.017**
Other arrhythmias	35 (25.7)	9 (34.6)	22 (22.2)	9 (25.0)	0.449
EPS performed	68 (50.0)	18 (69.2)	45 (45.5)	8 (22.2)	**0.001**
Inducible VT/VF*	59 (86.8)	16 (88.9)	38 (84.4)	7 (87.5)	1.00
24-h Holter	28 (20.6)	5 (19.2)	20 (20.2)	5 (13.9)	0.742
Abnormal Holter*	15 (53.6)	4 (80.0)	9 (45.0)	3 (60.0)	0.428
Mortality	7 (5.16)	0 (0)	6 (6.06)	3 (8.33)	0.427
Follow up period (months)	97.5 ± 61.8	118 ± 57.1	92.9 ± 61.9	85.1 ± 66.4	**0.043**

The brackets contain the percentage of patients fulfilling the criteria in the overall cohort, or within the respective subgroup. The P-value for categorical variables were obtained by Fisher’s exact test, and for continuous variables by Kruskal–Wallis rank test one-way ANOVA. *indicates that value indicates the patient percentage within the subgroup population where the investigation is performed. Bolded values indicate P < 0.05.

**TABLE 1B:** Baseline characteristics with significant intergroup differences.

Characteristic	Overall (*n* = 136)	Asymptomatic (*n* = 26)	Syncope (*n* = 99)	VT/VF (*n* = 36)	*P*-value		
					A vs. S	A vs. V	S vs. V
Family history SCD	14 (10.3)	5 (19.2)	9 (9.09)	0 (0)	0.166	**0.010**	0.112
EPS performed	68 (50.0)	18 (69.2)	45 (45.5)	8 (22.2)	**0.046**	**0.000**	**0.017**
Follow-up duration (months)	97.5 ± 61.8	118 ± 57.1	92.9 ± 61.9	85.1 ± 66.4	0.051	**0.010**	0.348

*A, asymptomatic; S, syncope; V, VT/VF. Bolded values indicate P < 0.05.*

The baseline ECG parameters are summarized in Table 2A. P-wave duration and R-wave amplitude in lead V5 differed significantly between the three subgroups (Table 2B). The syncope group had significantly longer P-wave duration than the asymptomatic group (*p* = 0.021). Moreover, R-wave amplitude in lead V5 differs significantly in the following descending order: asymptomatic, syncope, VT/VF (*P*-value: asymptomatic vs. syncope = 0.063, asymptomatic vs. VT/VF = 0.015, syncope vs. VT/VF = 0.034).

**TABLE 2A:** Baseline ECG parameter comparison.

ECG indices	Overall (*n* = 136)	Asymptomatic (*n* = 26)	Syncope (*n* = 99)	VT/VF (*n* = 36)	*P*-value
Heart rate (bpm)	79.4 ± 20.7	73.0 ± 10.8	79.8 ± 22.3	85.6 ± 28.3	0.355
P-wave duration (ms)	120 ± 15.9	109 ± 7.89	122 ± 16.7	119 ± 13.3	**0.044**
PR interval (ms)	173 ± 29.2	168 ± 22.5	175 ± 31.2	173 ± 31.6	0.534
QRS interval (ms)	112 ± 37.1	109 ± 17.3	113 ± 41.7	112 ± 28.4	0.911
QT interval (ms)	375 ± 52.2	383 ± 26.0	372 ± 54.8	379 ± 54.3	0.544
QTc (ms)	423 ± 39.3	419 ± 34.1	420 ± 38.4	435 ± 50.4	0.235
P-wave axis	59.9 ± 23.1	52.3 ± 19.3	61.0 ± 24.4	56.5 ± 32.3	0.127
QRS axis	50.2 ± 60.3	29.9 ± 44.8	52.4 ± 59.0	54.5 ± 83.2	0.219
T-wave axis	48.6 ± 32.8	42.3 ± 31.1	51.6 ± 33.4	45.8 ± 32.0	0.543
V1 S-wave amplitude (mV)	0.476 ± 0.288	0.387 ± 0.249	0.502 ± 0.288	0.397 ± 0.278	0.388
V5 R-wave amplitude (mV)	1.38 ± 0.551	1.70 ± 0.541	1.37 ± 0.515	1.13 ± 0.555	**0.012**

*Bolded values indicate P < 0.05.*

**TABLE 2B:** Baseline ECG parameter comparison with significant intergroup differences.

ECG indices	Overall (*n* = 136)	Asymptomatic (*n* = 26)	Syncope (*n* = 99)	VT/VF (*n* = 36)	*P*-value		
					A vs. S	A vs. V	S vs. V
P-wave duration (ms)	120 ± 15.9	109 ± 7.89	122 ± 16.7	119 ± 13.3	**0.021**	0.070	0.260
V5 R-wave amplitude (mV)	1.38 ± 0.551	1.70 ± 0.541	1.37 ± 0.515	1.13 ± 0.555	0.063	**0.015**	**0.034**

*Bolded values indicate P < 0.05.*

### Quinidine Use

In the present cohort, 29 patients were prescribed quinidine over the course of follow-up (asymptomatic = 2; syncope = 23; VT/VF = 14; mean single dose = 404 ± 163 mg; mean daily dose = 670 ± 297 mg; mean duration = 1573 ± 2178 days). The reasons for quinidine prescription were (1) recurrent VT/VF (*n* = 14); (2) recurrent PVC (*n* = 4); (3) VT/VF prophylaxis before ICD implantation or replacement (*n* = 6); (4) VT/VF prophylaxis for patients who initially refused ICD implantation (*n* = 2). Indications for quinidine use in three patients were not documented. Two patients were prescribed quinidine for recurrent VF, but the drug was withheld for observation of ICD shock frequency instead. Out of the 16 patients who were prescribed and given quinidine due to recurrent arrhythmia, 11 patients were treatment-responsive, 4 were non-responsive, and one patient was intolerant with symptoms of vertigo and numbness.

### Events During Follow-Up

The mean follow-up period is 97.5 ± 61.8 months (median = 95 months, interquartile range = 77.3 months), with 7 patients (mortality rate = 5.15%) passed away. The underlying causes of death, none of which was cardiogenic, include malignancy (*n* = 2), chronic obstructive pulmonary disease (*n* = 2), sepsis (*n* = 1), pneumonia (*n* = 1), and non-ST-elevation myocardial infarction (*n* = 1). Amongst the 68 patients (50.0%) who undertook EPS, 59 patients (20.6%) was found to be VT/VF inducible. Within the group of 28 patients (20.6%) who undergone 24-h Holter monitoring, rhythm abnormalities were found amongst 15 patients (53.6%). Insignificant intergroup differences were found in both the inducibility of EPS and the occurrence of abnormalities during 24-h Holter. Genetic testing was performed in 20.6% of the cohort (*n* = 28) and of these four patients tested positive for SCN5A mutation. All four patients belonged to the syncope group, and one patient is also a part of the VT/VF group.

For the baseline characteristics, significant inter-group differences were found in the family history of SCD, performance of EPS and follow-up period. Findings under further analysis are presented in Table 1B. Family history of SCD/VF was noted to be more commonly observed amongst the asymptomatic group than the VT/VF group (*p* = 0.017). Furthermore, the prevalence of EPS delivery differed significantly when the three subgroups undergone pairwise comparison, in descending order of prevalence: asymptomatic, syncope and VT/VF group (*P*-value: asymptomatic vs. syncope = 0.046; asymptomatic vs. VT/VF ≤ 0.0001; syncope vs. VT/VF = 0.017). Additionally, the follow-up period for the asymptomatic group is significantly longer than the VT/VF group (*p* = 0.010).

### Outcomes of ICD Therapy

In total, 252 ICD has been implanted (*n* = 252, average per patient = 1.85 ± 1.00), of which the vast majority is automatic (*n* = 238, 94.4%), and the remaining devices were subcutaneous (S-ICD) (*n* = 14, 5.56%). 51.5% of the cohort undergone ICD replacement at least once (*n* = 60, mean years before replacement = 5.06 ± 1.81). The outcomes of ICD therapy are presented in Table 3A. The overall complication rate is 22.1% (*n* = 30), with device and infection-related complications present in 9.56% of the cohort (*n* = 13), including 5 patients with lead malfunction (3.68%), 6 patients with lead dislocation (4.41%), and 3 patients with post-operative wound infection (2.21%). No significant inter-group differences were found for the complications. 6 patients required lead repositioning due to lead dislodgement or noise sensing, and 4 patients ultimately undergone ICD removal after initial implantation, and/or subsequent ICD replacement due to infection (*n* = 4), lead erosion (*n* = 1) and infective endocarditis (*n* = 1). S-ICD was implanted in 1 case of lead malfunction, 1 case of lead dislocation, and 2 cases of infection.

**TABLE 3A:** Outcomes of ICD therapy.

Outcome	Overall (*n* = 136)	Asymptomatic (*n* = 26)	Syncope (*n* = 99)	VT/VF (*n* = 36)	*P*-value
Appropriate shock	34 (25.0)	3 (11.5)	26 (26.3)	17 (47.2)	**0.007**
Inappropriate shock	24 (17.6)	5 (19.2)	16 (16.2)	8 (22.2)	0.676
Lead malfunction	6 (4.41)	1 (3.85)	5 (5.05)	2 (5.56)	1.00
Lead dislocation	5 (3.68)	0 (0)	5 (5.05)	2 (5.56)	0.736
Infection	3 (2.21)	1 (3.57)	2 (2.02)	0 (0)	0.514
Pre-ICD VT/VF episodes	0.437 ± 0.903	–	0.429 ± 0.919	1.64 ± 1.05	**0.000**
Post-ICD VT/VF episodes	4.24 ± 12.7	2.23 ± 5.46	4.29 ± 13.6	7.56 ± 15.8	**0.037**
Total VT/VF episodes	4.65 ± 12.7	2.23 ± 5.46	4.69 ± 13.6	9.19 ± 15.6	**0.000**

*Bolded values indicate P < 0.05.*

Appropriate shocks were executed in 25.0% of the cohort during the follow-up period (*n* = 34, male = 100%, age = 49.3 ± 15.2). Amongst these patients, 19 patients (55.9%) presented with type 1 pattern, and 11 patients (32.4%) experienced pattern evolution. It is worth noting that three patients (8.82%) were previously asymptomatic, and 17 patients (50.0%) did not experience VT/VF prior to ICD implantation. Amongst the 17 patients, two patients have relevant family history (BrS: *n* = 1; VF/SCD: *n* = 2), 9 out of the 1010 patients with EPS performed showed inducible VT/VFS, and 7 of the 10 with drug challenge performed were tested positive. Ten patients presented with type 1 BrP, and three of these patients experienced pattern evolution to a type 2 pattern. Patients with appropriate shocks experienced an average of 4.71 ± 12.8 VT/VF episodes in total.

**FIGURE 1:**
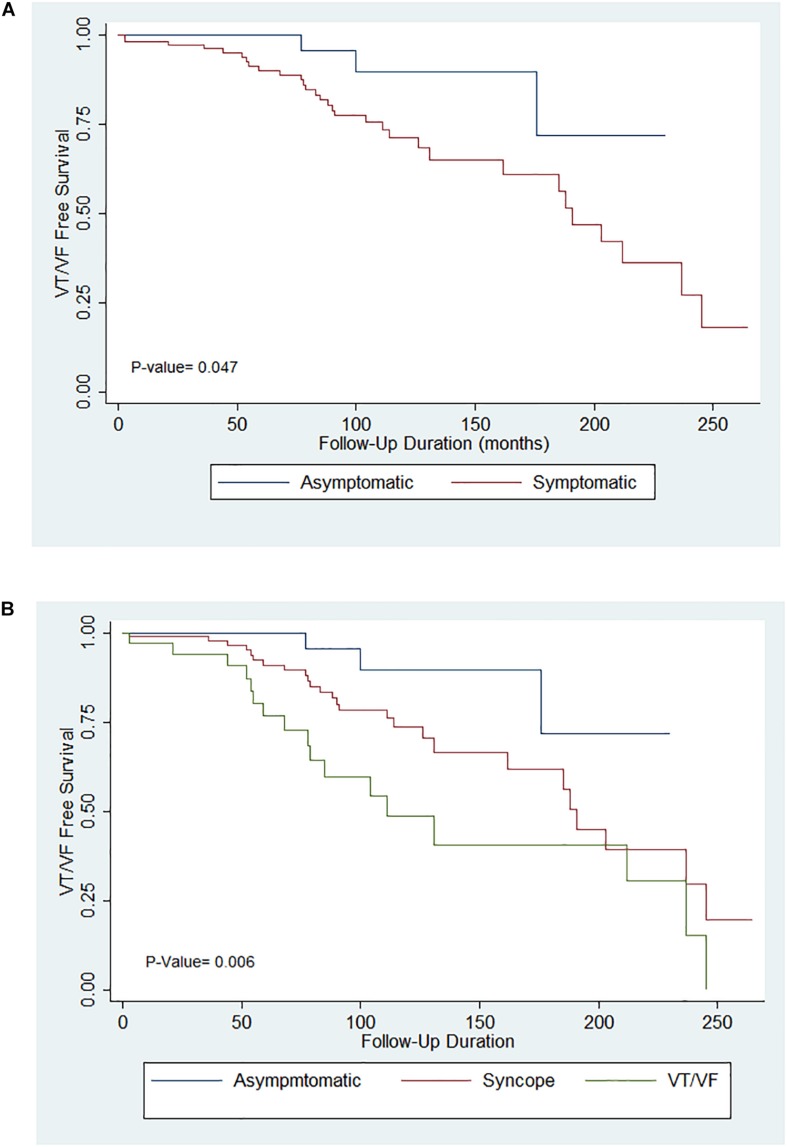
**(A)** Kaplan–Meier curves for appropriate implantable cardioverter-defibrillator (ICD) shocks in the asymptomatic and symptomatic groups. **(B)** Kaplan–Meier curves for appropriate ICD shocks in the asymptomatic, syncope and VT/VF groups.

Significant differences were found when comparing the delivery of appropriate shocks between initially asymptomatic and symptomatic patients (Figure 1A, *p* = 0.007). Further comparison of survival estimates between the three patient groups is presented on Figure 1B), where the VT/VF group is presented to be most likely to receive an appropriate shock, followed by the syncope group (*P*-value: asymptomatic vs. syncope = 0.062, asymptomatic vs. VT/VF = 0.003, syncope vs. VT/VF = 0.034). Table 3B shows that under pairwise comparison, the VT/VF group is found to be significantly more likely to experience appropriate shocks in comparison to both the asymptomatic and syncope group. Whilst it is expected for the number of VT/VF episodes prior to ICD implantation to be significantly higher in the VT/VF group, it is worth noting that the difference in number of VT/VF episodes between the asymptomatic and VT/VF group was not significant post implantation (*p* = 0.063). Significant difference was only found when comparing between the syncope and VT/VF group (*p* = 0.014). VT/VF group remains to have significantly higher number of total VT/VF episodes in comparison to the remaining two subgroups (*P*-value: asymptomatic vs. syncope = 0.365; asymptomatic vs. VT/VF ≤ 0.0001; syncope vs. VT/VF ≤ 0.0001).

**TABLE 3B:** Implantable cardioverter-defibrillator outcomes with significant intergroup differences.

Characteristic	Overall (*n* = 136)	Asymptomatic (*n* = 26)	Syncope (*n* = 99)	VT/VF (*n* = 36)	*P*-value		
					A vs. S	A vs. V	S vs. V
Appropriate shock	34 (25.0)	3 (11.5)	26 (26.3)	17 (47.2)	0.115	**0.003**	**0.021**
Post-ICD VT/VF episodes	4.24 ± 12.7	2.23 ± 5.46	4.29 ± 13.6	7.56 ± 15.8	0.971	0.063	**0.014**
Total VT/VF episodes	4.65 ± 12.7	2.23 ± 5.46	4.69 ± 13.6	9.19 ± 15.6	0.365	**0.000**	**0.000**

*Bolded values indicate P < 0.05.*

### Cox Regression Analysis

The results for the univariate Cox regression analysis of clinical predictors for the occurrence of appropriate shock in both the entire study cohort and the subgroups are presented in Table 4A. Concomitant presence of other arrhythmia is an independent predictor for appropriate shock delivery overall (*p* = 0.040, HR = 2.09, 95% CI = [1.03, 4.22]). None of the clinical characteristics showed statistical significance in their prediction for appropriate shocks for the asymptomatic group. The presentation of other arrhythmia is also predictive for the syncope group (*p* = 0.038, HR = 2.328, 95% CI = [1.05, 5.14]), while the male sex (*p* = 0.014, HR = 32.4, 95% CI = [2.02, 519]) and the presentation of type 1 BrP (*p* = 0.040, HR = 4.90, 95% CI = [1.07, 22.3]) are predictive for the VT/VF group. Given their statistical significance in the prediction of appropriate shock delivery, the three clinical characteristics: (1) male sex; (2) presentation of type 1 pattern; (3) concurrent presentation of other arrhythmia were included in the multivariate Cox regression analysis predictor model. However, as illustrated on Table 4B, type 1 BrP and the presence of other arrhythmia are significant predictors in the multivariate model (*P*-value: male = 0.674; type 1 pattern = 0.035; other arrhythmia = 0.041). The results of sensitivity analysis by excluding females are shown in Supplementary Tables 1A,B.

**TABLE 4A:** Univariate cox-regression analysis of appropriate shock predictors.

Clinical characteristic	*Z* score	Hazard ratio [95% confidence interval]	*P*-value
**Overall cohort**			
Male	−0.68	0.494 [0.064, 3.80]	0.498
Age of initial BrP presentation	−0.21	0.997 [0.974, 1.02]	0.832
BrP evolution	−0.21	0.925 [0.446, 1.92]	0.835
Type 1 BrP	1.10	1.51 [0.728, 3.12]	0.270
Fever-induced BrP	1.13	2.32 [0.542, 9.96]	0.256
Syncope	0.88	1.44 [0.644, 3.20]	0.376
Other arrhythmias	2.05	2.09 [1.03, 4.22]	**0.040**
Inducible VT/VF	−0.07	0.957 [0.272, 3.36]	0.945
**Asymptomatic**			
Male	−	−	−
Age of initial BrP presentation	0.63	1.03 [0.939, 1.13]	0.526
BrP evolution	0.57	2.24 [0.140, 35.9]	0.568
Type 1 BrP	−1.10	0.257 [0.023, 2.92]	0.273
Fever-induced BrP	−	−	−
Syncope	−	−	−
Other arrhythmias	0.43	1.85 [0.115, 29.9]	0.665
Inducible VT/VF	−	−	−
**Syncope**			
Male	0.00	0.00	1.00
Age of initial BrP presentation	−0.52	0.993 [0.965, 1.02]	0.601
BrP evolution	−0.27	0.892 [0.392, 2.03]	0.786
Type 1 BrP	1.68	2.09 [0.882, 4.97]	0.094
Fever-induced BrP	0.22	1.25 [0.165, 9.50]	0.828
Syncope	−	−	−
Other arrhythmias	2.07	2.32 [1.05, 5.14]	**0.038**
Inducible VT/VF	1.20	3.88 [0.427, 35.2]	0.228
**VT/VF**			
Male	2.46	32.4 [2.02, 519]	**0.014**
Age of initial BrP presentation	0.18	1.00 [0.973, 1.03]	0.860
BrP evolution	0.87	1.61 [0.555, 4.66]	0.382
Type 1 BrP	2.05	4.90 [1.07, 22.3]	**0.040**
Fever-induced BrP	0.92	2.04 [0.450, 9.22]	0.356
Syncope	−0.09	0.952 [0.323, 2.80]	0.929
Other arrhythmias	0.31	1.19 [0.407, 3.46]	0.754
Inducible VT/VF	−	−	−

*Bolded values indicate P < 0.05.*

**TABLE 4B:** Multivariate cox-regression analysis appropriate shock predictors.

Feature	*Z* score	Hazard ratio [95% confidence interval]	*P*-value
Male	−0.42	0.644 [0.083, 4.99]	0.674
Type 1 BrP	2.11	2.07 [1.05, 4.06]	**0.035**
Other arrhythmias	2.04	1.92 [1.03, 3.58]	**0.041**

*Bolded values indicate P < 0.05.*

17.6% (*n* = 24) of the cohort experienced at least one inappropriate shock. The reasons underlying inappropriate shocks include SVT (*n* = 10), AF (*n* = 8), noise-sensing (*n* = 2), T-wave oversensing (*n* = 2), and transcutaneous electrical nerve stimulation (*n* = 1). Intergroup differences for the delivery of inappropriate shocks were statistically insignificant (*p*-value = 0.676). Given most cases of inappropriate shocks reported in this cohort is related to concomitant arrhythmias, it was found to significantly increase the risk of inappropriate shock (*p* ≤ 0.0001, HR = 5.09, 95% CI = [2.17, 11.9]). The univariate Cox proportional hazard ratio analysis of the clinical characteristic for the delivery of inappropriate shocks is summarized in Table 5. Amongst patients with inappropriate shock delivered, 41.7% (*n* = 10) received appropriate shocks as well. The results of sensitivity analysis by excluding females are shown in Supplementary Table 2.

**TABLE 5:** Univariate cox-regression analysis inappropriate shock predictors.

Feature	*Z* score	Hazard ratio [95% confidence interval]	*P*-value
Age	0.23	1.00 [0.975, 1.03]	0.821
BrP evolution	0.57	1.28 [0.550, 2.97]	0.568
Syncope	−0.33	0.864 [0.365, 2.04]	0.739
Other arrhythmia	3.75	5.09 [2.17, 11.9]	**0.000**

*Bolded values indicate P < 0.05.*

## Discussion

Implantable cardioverter-defibrillator implantation is an effective therapy for the prevention of SCD in BrS patients. However, it is also associated with complications, and thus routine prophylactic use of ICDs in all BrS patients are not currently recommended. In this study, we conducted a retrospective analysis of a large cohort of BrS patients receiving ICDs with a median follow-up of 95 months. At least one appropriate shock is delivered to 25.0% of the cohort, with an ICD-related adverse event rate of 22.1%. Complications include inappropriate shock delivery, lead malfunction, lead dislodgement and post-operative infection. Similar rates in appropriate therapy and complications have been reported by other studies, which highlights the importance of risk-benefit analysis prior to ICD implantation (Conte et al., 2015; Dereci et al., 2019). It should be noted that none of the deceased patients had a cardiogenic cause of death, which reflects the effectiveness of ICD in the prevention of SCD.

The importance of ICD implantation in the secondary SCD prevention is well-demonstrated with 47.2% patients in the VT/VF group experienced appropriate shocks, the highest percentage in comparison to the asymptomatic and syncope group. Similar rates of appropriate shock for secondary prevention of SCD have been previously reported (Sacher et al., 2013; Conte et al., 2015). Furthermore, the VT/VF group were found to have a significantly greater number of mean total VT/VF episodes (*n* = 9.19 ± 15.6 episodes, *p* ≤ 0.0001). Hence, strong evidence supports ICD implantation in patients with a history of VT/VF due to their increased likelihood for recurrence of VT/VF. Moreover, 26.3% of the syncope group (*n* = 26), of which 46.2% were VT/VF-free prior to implantation (*n* = 12), received appropriate shocks. Whilst this result illustrates a greater rate of appropriate shocks amongst symptomatic BrS patients, difficulty in the differentiation between cardiogenic and non-cardiogenic syncope should be noted. Of note, 63.0% patients that received appropriate shocks were VT/VF-naïve prior to ICD implantation. Three patients were asymptomatic and has no family history of SCD, which is 11.5% of the asymptomatic group, suggesting that ICD implantation in asymptomatic patient remains controversial (Antzelevitch et al., 2005). The difference in the number of post-implantation VT/VF episodes is statistically insignificant between the asymptomatic and VT/VF group, which highlights the importance in continuous follow-up and the potential underestimation of arrhythmic risk in asymptomatic patients.

Given the multifactorial nature of the SCD risk amongst BrS patients, the significance of a multiparametric approach to improve the risk stratification has been explored in the recent years (Sieira et al., 2017a). The present study highlights the need to identify better predictors for VT/VF, and contributes to the identification of key risk factors. Whilst type 1 BrP is recognized to be of higher arrhythmic risk, the dynamicity in BrP can result in missed diagnosis of the pattern (Richter et al., 2009). Recently, it has been reported that increased temporal variability was observed in type 1 BrP, suggesting that serial ECG and 24-h Holter studies can be of prognostic value (Gray et al., 2017; Castro Hevia et al., 2019). Electrocardiographic markers may be useful in risk stratification, particularly amongst the asymptomatic patients (Morita et al., 2008; Letsas et al., 2017). Despite recent studies have reported an association between inducible EPS and increased risk of arrhythmia, the predictive value of EPS remains controversial (Sieira et al., 2017b; Li et al., 2018; Yuan et al., 2018). Previously, it was found that when combined with a spontaneous type 1 pattern, a positive EPS study was associated with a worse prognosis (Li et al., 2018). In the present study, EPS inducibility was not raised in the VT/VF group, and inducible EPS was not predictive of appropriate shock delivery overall or for individual subgroups. Although there is increasing understanding in the genetic mechanisms underlying BrS, the low prevalence of genetic testing prevents the involvement of genetics in BrS risk stratification at least in our locality. Large-scale genetic testing upon patient cohorts is needed to gain insights on the clinical role of genetics in BrS.

The delivery of inappropriate shocks remains to be the most common complication in ICD implantation. The rate of inappropriate shocks reported in the presents study (17.6% at 12-years follow up) is consistent with data previously reported by other groups, ranging from 15% at 8-years follow up to 24% at 6-years follow up (Steven et al., 2011; Sacher et al., 2013). At least one inappropriate shock occurred in 18.7% patients at 20-year follow-up of the single-center cohort that was the first to report BrS (Conte et al., 2015). Although the concomitant presence of other arrhythmia often underlies the delivery of inappropriate shocks, the concurrent presentation of other arrhythmia was not found to increase the risk of inappropriate shock across all patient groups. However, numerous studies have reported AF to be a predictor of inappropriate shock delivery, possibly due to rapid ventricular response to the atrial impulse and more advanced disease progression (van Gelder et al., 2011; van Rees et al., 2011; Mustafa et al., 2018). Catheter ablation may lower the chance of inappropriate shock delivery in patients presented with AF (Prabhu et al., 2017). Catheter ablation were used to manage symptomatic BrS amongst a small number of cases, with techniques including epicardial mapping, endocardial mapping and VF-triggering premature ventricular complex applied (Fernandes et al., 2018). Higher VF zone threshold (>200 beats per minute) and delayed therapy at pre-VF zone (>170 beats per minute) can help to reduce SVT-related inappropriate shocks (Moss et al., 2012). It is worth noting that 10 patients received both appropriate and inappropriate shocks, suggesting that risk factors for inappropriate shock should not contraindicate ICD implantation.

Device and infection-related complications is relatively low in the present cohort. Unlike the France, Italy, Netherlands, Germany BrS registry, which reported a 29% risk of post-implantation lead failure within 10-years, only 6 patients experienced lead malfunction in the present study. The large difference in rate of lead malfunction is likely due to earlier ICD replacement (mean years = 5.06 ± 1.81) in the present cohort. S-ICD is an emerging alternative to the traditional automatic ICD. Recent studies reported comparable or reduced complication rate in S-ICD (Baalman et al., 2018; Chen C. F. et al., 2019). In the present cohort, S-ICDs were implanted in younger patients (mean initial presentation age = 42.3 ± 18.4) or patients with greater infection risk (Viani et al., 2019). A larger cohort is needed to evaluate the efficacy and safety of S-ICD compared to the conventional automatic ICD.

### Limitations

Several limitations should be noted for the present study. Firstly, due to the multicenter nature of the present study, inconsistency may be present in data collection despite our best effort. Secondly, since this was a retrospective study, ICD technology and their settings have evolved over the course of follow-up. ICD programming may have differed between centers, and the definition of sustained VT/VF requiring ICD shock was determined by individual physicians who entered the case records without standardization. Thirdly, one patient was lost to follow-up. Fourthly, the use of appropriate shock as a surrogate for SCD may result in an overestimation of the death risk. Finally, we were not able to access data stored on the ICDs, such as program settings and ECG waveforms, but relied on case records written by the physicians. Future studies can use stored ICD data to determine the frequency and occurrence of pattern conversion between types 1 and 2, as well as correlation between appropriate shocks and conversion into type 1 close to the event.

## Conclusion

The present study demonstrates that ICD therapy is effective in both primary and secondary prevention of SCD in BrS patients. The VT/VF group is most likely to receive appropriate shocks, followed by the syncope group and lastly the asymptomatic group. However, the asymptomatic group experienced comparable number of VT/VF episodes post-implantation, which highlights the importance of careful continuous monitoring in all patient populations. Currently, there is a lack of clinical predictors for both appropriate and inappropriate ICD therapy delivery. Further research on electrocardiographic and genetic markers may aid in the risk-benefit analysis for ICD implantation in patients with Brugada ECG patterns.

## Data Availability Statement

All datasets generated for this study are included in the article/Supplementary Material.

## Ethics Statement

This retrospective study received Ethics approval from The Joint Chinese University of Hong Kong – New Territories East Cluster Clinical Research Ethics Committee and is based on datasets that have already been made available in an online repository.

## Author Contributions

SL and GT contributed to the conception, design, analysis, interpretation, manuscript drafting, and manuscript revision. SL, KHL, JZ, KSL, RL, GL, TL, KPL, NM, QZ, and GT contributed to the data interpretation and manuscript revision.

## Conflict of Interest

The authors declare that the research was conducted in the absence of any commercial or financial relationships that could be construed as a potential conflict of interest.
