# Supplementary material for: Outcomes in Brugada Syndrome Patients With Implantable Cardioverter-Defibrillators: Insights From the SGLT2 Registry
Source: Front Physiol. 2020 Mar 10;11:204. doi: 10.3389/fphys.2020.00204 (PMC7076170; doi:10.3389/fphys.2020.00204)
Supplement: Supplementary file 1 [file Table_1.DOCX]

Supplementary Table 1. Univariate Cox-Regression Analysis of Appropriate Shock Predictors (sensitivity analysis by excluding females)

| Clinical Characteristics | Z Score | Hazard Ratio  [95% Confidence Interval] | P-value |
| --- | --- | --- | --- |
| *Overall Cohort* | | | |
| Age of Initial BrP Presentation | 0.32 | 1.00 [0.981, 1.03] | 0.745 |
| BrP Evolution | -0.41 | 0.854 [0.402, 1.82] | 0.683 |
| Type 1 BrP | 0.95 | 1.43 [0.684, 2.97] | 0.344 |
| Fever-induced BrP | 0.17 | 1.19 [0.160, 8.88] | 0.865 |
| Syncope | 1.19 | 1.67 [0.717, 3.88] | 0.235 |
| Other Arrhythmias | 1.78 | 1.92 [0.934, 3.95] | 0.076 |
| Inducible VT/VF | 0.02 | 1.01 [0.288, 3.57] | 0.983 |
| *Asymptomatic* | | | |
| Age of Initial BrP Presentation | 0.63 | 1.03 [0.939, 1.13] | 0.526 |
| BrP Evolution | 0.57 | 2.24 [0.140, 35.9] | 0.568 |
| Type 1 BrP | -1.10 | 0.257 [0.023, 2.92] | 0.273 |
| Fever-induced BrP | - | - | - |
| Syncope | - | - | - |
| Other Arrhythmias | 0.43 | 1.85 [0.115, 29.9] | 0.665 |
| Inducible VT/VF | - | - | - |
| *Syncope* | | | |
| Age of Initial BrP Presentation | 0.11 | 1.00 [0.973, 1.03] | 0.911 |
| BrP Evolution | 0.29 | 1.13 [0.495, 2.57] | 0.774 |
| Type 1 BrP | 1.38 | 1.83 [0.777, 4.30] | 0.167 |
| Fever-induced BrP | 0.17 | 1.19 [0.157, 9.01] | 0.867 |
| Syncope | - | - | - |
| Other Arrhythmias | 1.77 | 2.07 [0.927, 4.61] | 0.076 |
| Inducible VT/VF | 0.65 | 2.01 [0.242, 16.7] | 0.518 |
| *VT/VF* | | | |
| Age of Initial BrP Presentation | 0.81 | 1.01 [0.982, 1.04] | 0.417 |
| BrP Evolution | 0.52 | 1.35 [0.438, 4.15] | 0.602 |
| Type 1 BrP | 1.97 | 4.64 [1.01, 21.4] | **0.049** |
| Fever-induced BrP | 0.06 | 1.06 [0.137, 8.27] | 0.953 |
| Syncope | 0.29 | 1.19 [0.369, 3.81] | 0.775 |
| Other Arrhythmias | 0.31 | 1.19 [0.407, 3.46] | 0.754 |
| Inducible VT/VF | - | - | - |

Supplementary Table 1B. Multivariate Cox-Regression Analysis Appropriate Shock Predictors (sensitivity analysis by excluding females)

| Feature | Z Score | Hazard Ratio  [95% Confidence Interval] | P-Value |
| --- | --- | --- | --- |
| Type 1 BrP | 1.16 | 1.56 [0.734, 3.31] | 0.248 |
| Other Arrhythmias | 1.91 | 2.04 [0.981, 4.24] | 0.056 |

Supplementary Table 2. Univariate Cox-Regression Analysis Inappropriate Shock Predictors (Exclude Female)

| Feature | Z Score | Hazard Ratio  [95% Confidence Interval] | P-Value |
| --- | --- | --- | --- |
| Age | 0.91 | 1.01 [0.985, 1.04] | 0.365 |
| BrP Evolution | 0.33 | 1.16 [0.485, 2.78] | 0.738 |
| Syncope | 0.02 | 1.01 [0.410, 2.48] | 0.986 |
| Other Arrhythmias | 3.51 | 4.70 [1.98, 11.2] | **0.000** |
